# Safety and Efficacy of Stent-assisted Coiling in the Treatment of Unruptured Wide-necked Intracranial Aneurysms: A Single-center Experience

**DOI:** 10.7759/cureus.4847

**Published:** 2019-06-06

**Authors:** Pedro Aguilar-Salinas, Leonardo B Brasiliense, Roberta Santos, Gustavo Cortez, Douglas Gonsales, Amin Aghaebrahim, Eric Sauvageau, Ricardo A Hanel

**Affiliations:** 1 Neurosurgery, Banner University Medical Center Tucson, Tucson, USA; 2 Neurosurgery, University of Arizona, Tucson, USA; 3 Lyerly Neurosurgery, Baptist Neurological Institute, Jacksonville, USA

**Keywords:** intracranial aneurysms, aneurysm occlusion, coiling, stent-assisted, wide-necked

## Abstract

Introduction: Wide-necked intracranial aneurysms (IAs) are complex lesions that may require different microsurgical or endovascular strategies, and stent-assisted coiling (SAC) has emerged as a feasible alternative to treat this subset of aneurysms.

Methods: The objective was to assess the rate of complications of unruptured wide-necked IAs treated with SAC. We retrospectively identified patients with unruptured wide-necked IAs treated with SAC. Medical charts, procedure reports, and imaging studies were analyzed.

Results: One hundred twenty patients harboring 124 unruptured wide-necked IAs were included. Ninety-two aneurysms (74.2%) were located in the anterior circulation. The median aneurysm size was 7 mm (IQR = 5-10). The immediate complete aneurysm occlusion rate was 29% (36/124). The rate of procedural complications was 3.3 % (4/120), which included 2 intraprocedural aneurysm ruptures, 1 immediate postprocedure aneurysm rupture, and 1 vessel occlusion rescued with an open-cell stent. The median follow-up time was 21 months (IQR = 10.3-40.9). Kaplan-Meier analysis estimated a median time of complete aneurysm occlusion of 6.3 months (95%CI = 3.8-7.8). At 30-day follow-up, 80.7% of patients had a Glasgow Outcome Score (GOS) of 5 and at the latest follow-up 83.9%. Imaging follow-up was available for 102 patients. The rate of complete aneurysm occlusion was 73.5% (75/102), severe in-stent stenosis (>50%) was found in 1% (1/102), the recanalization rate was 6.6% (5/75), and the retreatment rate was 7.8% (8/102).

Conclusion: SAC remains a safe and effective technique to treat wide-necked IAs, providing a low rate of complications and recanalization with excellent long-term aneurysm occlusion rates.

## Introduction

Since the early 1990s, with the introduction of detachable coils by Guglielmi, neurovascular treatment of aneurysms has seen widespread development of improved techniques, overcoming previous limitations, and increasingly moving towards less invasive strategies [1]. In the setting of wide-necked aneurysms, microsurgical clipping has long been considered the definitive treatment; however, not all aneurysms are surgically accessible without risks of serious morbidity. Primary coiling is a feasible technique for aneurysms with a favorable dome-to-neck ratio (2 or greater). Stent-assisted coiling (SAC) was developed for aneurysms with a neck diameter of 4 mm or greater, to provide mechanical support and prevent coil prolapse into the lumen as well as a scaffold for endothelialization, facilitating aneurysm thrombosis [2-5]. Regardless of the SAC technique, reported complications include vessel perforation, thromboembolic events, in-stent stenosis, and hemorrhage.

In this article, we present our experience with SAC embolization in patients with unruptured wide-necked intracranial aneurysms at a high-volume center and assess the rate of procedure technical events, long-term aneurysm occlusion, in-stent stenosis, and retreatment.

## Materials and methods

Study design 

This study received IRB approval prior to data collection, written informed consent was not required due to the retrospective nature of the study, and a waiver of HIPAA privacy authorization was obtained. We retrospectively reviewed our database to identify patients with unruptured wide-necked aneurysms. Wide-necked aneurysm was defined as a dome-to-neck ratio < 2 or neck size > 4 mm. We included patients who underwent elective SAC between August 2007 and February 2014. Charts were reviewed to obtain initial clinical evaluations, procedure reports, hospital stay, aneurysm characteristics, as well as clinical and angiographic follow-ups. A single operator with assistant performed all interventions. The occlusion aneurysm rate was evaluated with digital subtraction angiography (DSA) and magnetic resonance angiogram (MRA) based on the Raymond-Roy occlusion classification [6]. Our primary outcome was to assess the rate of procedure technical events. Our secondary outcomes were to assess the rate of complete aneurysm occlusion, in-stent stenosis, aneurysm recanalization, and retreatment based on the latest imaging follow-up available. In addition, the Glasgow Outcome Scale (GOS) was used to determine postoperative functional outcomes at 30-day and latest clinical follow-up available. Recanalization was defined as aneurysm filling progression from Raymond-Roy class 1 to class 2 or 3 and from 2 to 3. Clinical follow-up was typically scheduled at 30 days, 3-6 months, and yearly thereafter. Imaging follow-up for unruptured aneurysms was typically scheduled at 6 months, 18 months, and every 3 years. For ruptured aneurysms, it was usually scheduled at 3 months, 6 months, 12 months, 24 months, and every 3 years. 

Endovascular treatment

**Figure 1:**
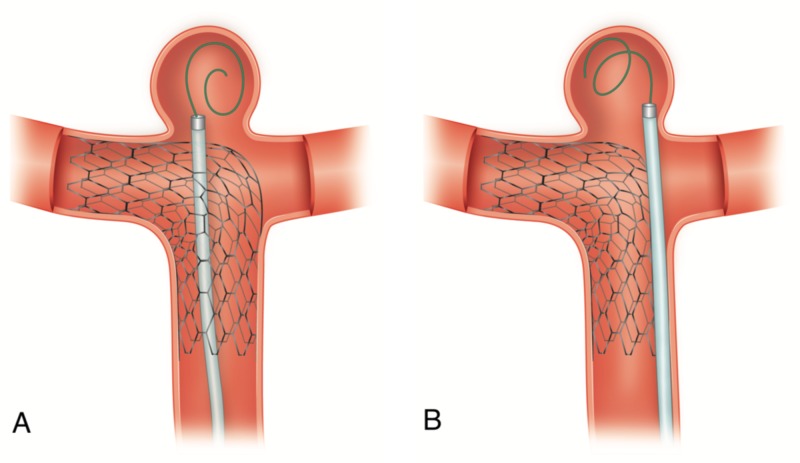
Techniques employed in the study for stent-assisted coiling Stent-assisted coiling embolization techniques. (A) Coil-through struts technique; the stent is fully deployed across the neck of the aneurysm and the microcatheter is navigated through the struts to detach coils into the sac of the aneurysm. B) Jailing technique; the sent is deployed after the microcatheter is placed inside the sac of the aneurysm and coiling detachment is performed once the stent is in place.

Patients were started on dual-antiplatelet therapy with aspirin (325 mg/day) and a thienopyridine derivative (clopidogrel, 75 mg daily or ticagrelor, 90 mg BID) at least five to seven days before the procedure, based on our previously published P2Y12-Reactive Units (PRU) protocol [7-8]. Interventions were performed using either local anesthesia or under general anesthesia. Intravenous heparin was systematically infused to maintain an activated clotting time greater than 200 seconds. The technique and choice of the stent were determined on a case-by-case basis by the surgeon (Figure 1). The Enterprise stent (Cordis Neurovascular, Miami, FL), the Neuroform stent (Stryker Neurovascular, Fremont, CA), the Liberty stent (Penumbra, Alameda, CA), and the LVIS Jr stent (MicroVention, Tustin, CA) were used. Dual-antiplatelet therapy was maintained for 3 months, followed by aspirin alone continued indefinitely. 

Statistical analysis

Descriptive data were presented as mean and standard deviation (SD) or median and interquartile range (IQR) for continuous variables according to data distribution, and absolute values and percentages for categorical variables. Time-to-event analysis was calculated by the Kaplan-Meier method to analyze the cumulative incidence and 95% confidence interval (CI) of complete aneurysm occlusion over time of all treated aneurysms after the SAC. Censored data were considered near complete, partial occlusion, and lost to follow-up. The analysis was performed with Stata software, version 14 (StataCorp, College Station, TX).

## Results

Demographics and aneurysms

**Figure 2:**
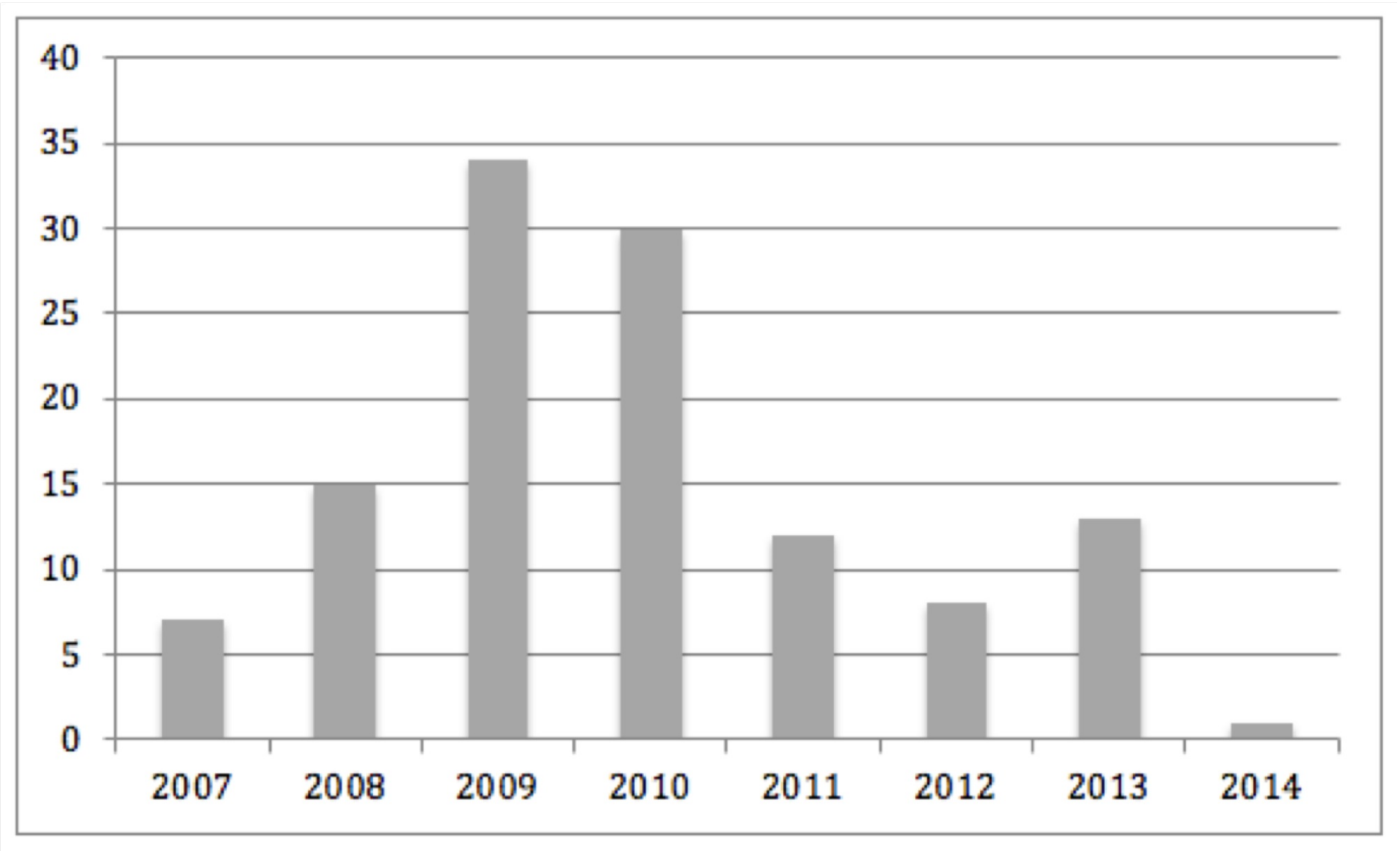
Distribution of patients with an unruptured wide-necked aneurysm treated per year

A total of 120 patients were included with 124 unruptured wide-necked aneurysms. The distribution of patients treated per year showed that 71.1% (86/120) were treated in the period from 2007 to 2010 (Figure 2).

There were 86 females (71.7%) and 34 males (28.3%) with a mean age of 62.6 years (SD = 12.7). The median aneurysm size was 7 mm (IQR = 5-10). Of the 124 aneurysms, 92 (74.2%) were located in the anterior circulation. The anterior communicating artery was the most frequent location, representing 25% of the sample (31/124). In 98.3% (118/120) of the cases, the stents were successfully deployed in the first attempt. One stent was required for 112 (90.3%) aneurysms and two stents were required for 12 (9.7%) aneurysms. The mean number of coils used per aneurysm was 6 (SD= 5.8) with a range from 1 to 40. Complete aneurysm occlusion immediately after the procedure was achieved in 29% (36/124), near complete occlusion 66.1% (82/124), and partial occlusion in 4.8% (6/124). Baseline characteristics are summarized in Table 1.

**Table 1:** Summary of presentation, size, and distribution of aneurysms AComm, anterior communicating; PComm, posterior communicating; ICA, internal carotid artery; PCA, posterior cerebral artery; PICA, posterior inferior cerebellar artery; ACA, anterior cerebral artery; MCA, middle cerebral artery.

Baseline characteristics of 124 unruptured wide-necked aneurysms	
Presentation (%)	
Incidental	92 (74.2)
Recurrence after coiling	8 (6.5)
Cranial neuropathy	6 (4.9)
Transient ischemic attack	2 (1.6)
Recurrence after clipping	2 (1.6)
Other	14 (11.2)
Aneurysm size (%)	
Small < 7 mm	60 (48.4)
Medium 7 - 12 mm	49 (39.5)
Large 13 - 24 mm	12 (9.7)
Giant > 25 mm	3 (2.4)
Circulation (%)	
Anterior	92 (74.2)
Posterior	32 (25.8)
Location (%)	
AComm	31 (25)
Basilar tip	19 (15.3)
ICA-Ophthalmic	18 (14.5)
ICA-Superior Hypophyseal	13 (10.5)
ICA-Cavernous	11 (8.9)
PComm	7 (5.6)
ICA-Terminus	5 (4)
Vertebral	4 (3.2)
PCA	4 (3.2)
PICA	3 (2.4)
ICA-Supraclinoid	2 (1.6)
Basilar trunk	2 (1.6)
ACA	2 (1.6)
MCA	2 (1.6)
ICA-Paraclinoid	1 (0.8)

Primary outcome

The overall procedure-technical event rate was 3.3% (4/120). Intracranial hemorrhage occurred in two patients due to intraprocedural rupture of the aneurysm. A patient developed a subarachnoid hemorrhage immediately after the procedure and died 4 days later. A vessel occlusion occurred while stent-assisted coiling an aneurysm in the posterior inferior cerebellar artery and an Enterprise stent was deployed to rescue with no final stroke-like symptoms nor any major neurological complication. In our series, there were no procedural thromboembolic complications. Table 2 provides more detailed information.

**Table 2:** Summary of procedure technical events MCA, middle cerebral artery; PICA, posterior inferior cerebellar artery; GOS, Glasgow outcome score; FU, follow-up.

Case	Event	Aneurysm location	Aneurysm size (mm)	Coils	Technique	Comments / Last follow-up
1	Postprocedure aneurysm rupture	Vertebro-basilar junction	28	4	Coil-through	Deceased 4 days after the procedure
2	Aneurysm Rupture	Left MCA	7	4	Jailing	48 months FU. Last GOS = 5. Complete aneurysm occlusion
3	Aneurysm Rupture	Right ICA-Ophthalmic	10	11	Jailing	Lost to FU
4	Vessel occlusion – stent used to open	Left PICA	9	3	Jailing	No-stroke like symptoms during hospitalization. 45 months FU. Last GOS = 5 and complete aneurysm occlusion.

Secondary outcomes

**Figure 3:**
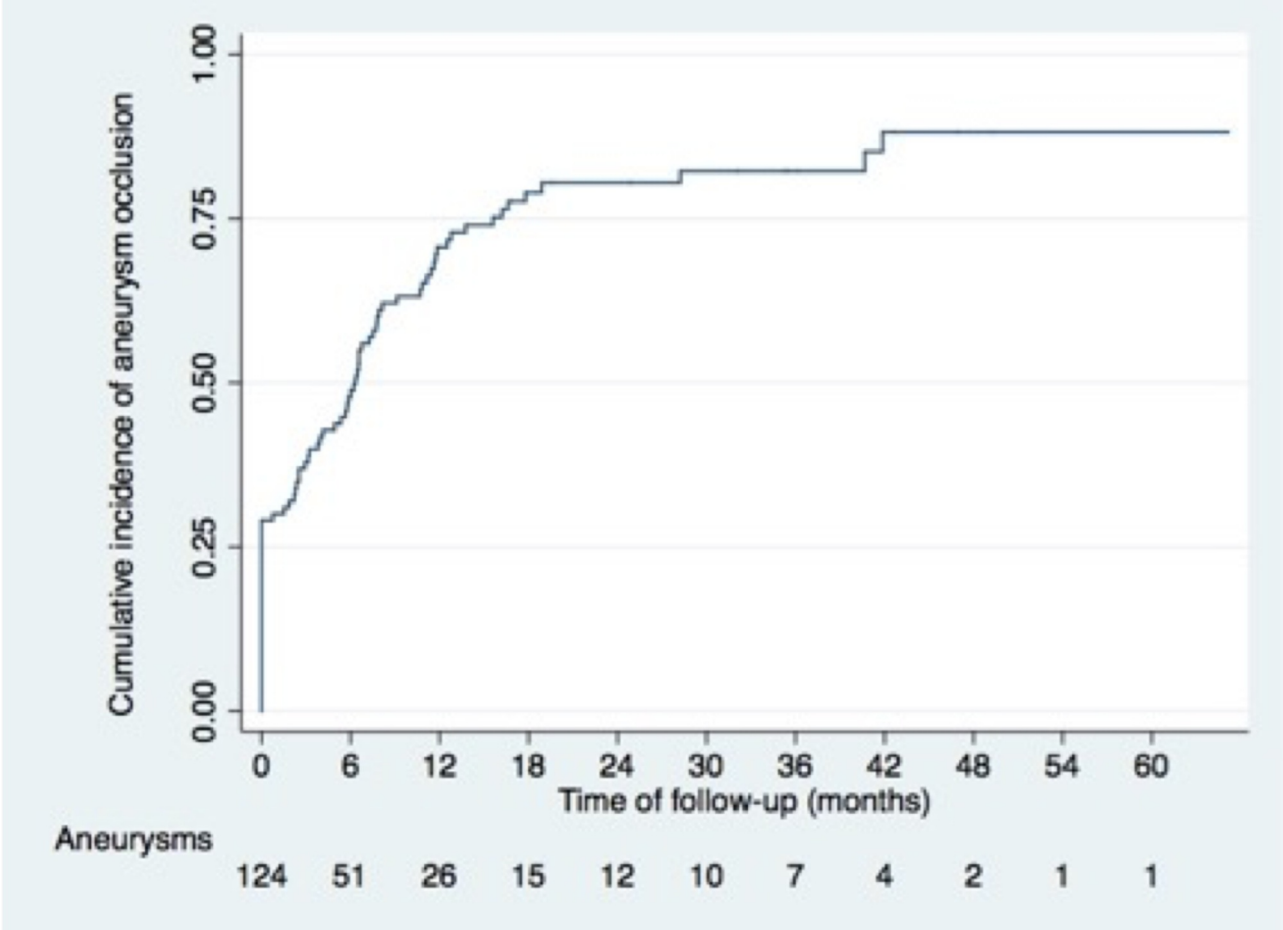
Time to complete aneurysm occlusion with Kaplan-Meier analysis

According to the Kaplan-Meier analysis, the median time for complete aneurysm occlusion was 6.3 months (95% CI = 3.8-7.8). The initial cumulative incidence for complete occlusion estimated for all aneurysms after treatment was 31.7% (95% CI = 24.1-41%). The cumulative incidences for complete aneurysm occlusion were 71.4% (95% CI = 62.5-79.8%), 79.6% (95% CI = 71-87%), 81% (95% CI = 72.4-88.3%), 82.7% (95% CI = 74.1-89.8%), and 88.5% (95% CI = 78.6-95.2%) at 12, 18, 24, 36, and 48 months, respectively (Figure 3).

At 30-day clinical follow-up, 80.7% of patients had a GOS of 5 and at the latest clinical follow-up, the proportion was 83.9%. Within the study period we found that 6 out of 7 deaths were not procedure-related, 4 of unknown cause, 1 of advanced-stage cancer, and 1 of cardiac disease. Eighteen patients were lost to imaging follow-up. Therefore, only 102 out of 120 patients (85%) were available to evaluate the rate of in-stent stenosis, recanalization, and retreatment. The median follow-up time was 21 months (IQR = 10.3 - 40.9). Based on the latest imaging follow-up, the rate of complete aneurysm occlusion was 73.5% (75/102). The rate of mild and severe (>50%) in-stent stenosis was 2.9% (3/102) and 1% (1/102), respectively. The rate of recanalization was 6.6% (5/75). Two out of 5 aneurysms evolved to Raymond-Roy class 3 and 2 evolved to class 3. The median time to recanalization was 13.7 months (IQR = 7.5-33.2). The overall rate of retreatment was 7.8% (8/102) with a median of 10.2 months (IQR = 2.3-20.6) after the first procedure was performed. Table 3 depicts detailed information. 

**Table 3:** Summary of cases retreated ICA, internal carotid artery; AComm, anterior communicating; PCA, posterior cerebral artery.

Case	Aneurysm location	Aneurysm size (mm)	Coils	Technique	Immediate Aneurysm Filling (Raymond-Roy classification)	Cause of retreatment	Time to retreatment (months)
1	ICA-Cavernous	20	10	Coil-Through	2	Incomplete occlusion	18.5
2	Basilar tip	12	9	Coil-Through	2	Incomplete occlusion	5.9
3	AComm	5	7	Coil-Through	1	Recanalization	27.7
4	ICA-Cavernous	34	30	Coil-Through	1	Recanalization	21.2
5	ICA-Cavernous	16	17	Jailing	2	Incomplete occlusion	13.8
6	Vertebral	10	16	Jailing	2	Incomplete occlusion	1.1
7	ACom	14	9	Coil-Through	2	Severe stent-stenosis	6.5
8	PCA	12	6	Coil-Through; Jailing	2	Incomplete occlusion	< 24 hours

## Discussion

Since the first successful report of SAC, the technique and devices have considerably improved, especially with the development of self-expanding intracranial stents [9-13]. Yet, endovascular treatment of wide-necked aneurysms remains a challenging procedure since a wide neck is considered a predictor of complications and aneurysm recurrence [14]. In this regard, the rate of procedure-related complications has been reported to range from 1% to 19% with thromboembolic events as the most frequent event, ranging from 4.3% to 9%, and mortality rates ranging from 1.4% to 8.7% [4,14-18]. However, it is important to underscore that most case series have reported outcomes in a heterogeneous sample with ruptured and unruptured wide-necked aneurysms and thus it remains difficult to establish the safety of SAC with unruptured aneurysms. Few studies evaluated SAC in the setting of elective treatment with the primary endpoint of analyzing safety with a specific stent and short/midterm occlusion results [5,15,19]. For this reason, we evaluated this endovascular technique as an elective treatment for unruptured wide-necked aneurysms. In our study, despite the variety of SAC techniques and stents, we found a low rate of procedure technical events (3.3%) and all-cause mortality (0.8%). The events included one vessel occlusion that was reopened with an Enterprise stent without neurological complications, 2 aneurysms that ruptured during coiling, and 1 aneurysm that ruptured immediately after the procedure causing the death of the patient from subarachnoid hemorrhage. Geyik et al. presented similar results after using different self-expanding stents to treat 500 aneurysms with SAC [20]. They reported an overall mortality rate of 1.9% but only 0.8% were procedure-related. The overall rate of thromboembolic events was 5.6%, which was attributed to the inclusion of both ruptured and unruptured aneurysms. The lack of thromboembolic events in our series is likely due to effective dual-antiplatelet therapy prior to the procedures. Thereby, our results confirm the safety of SAC in the setting of elective treatment at a high-volume center.

**Figure 4:**
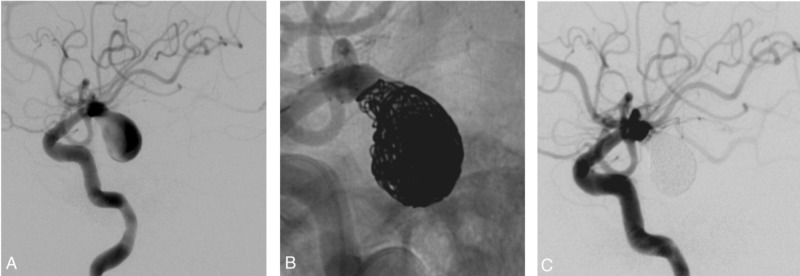
Female patient in her 80s treated with stent-assisted coil embolization for an unruptured wide-necked aneurysm. (A) Angiogram demonstrated a giant wide-necked aneurysm in the posterior communicating artery. (B) Immediate angiogram after the procedure showing dense coil packing of the aneurysm and placement of the stent over the parent artery. (C) Six-month follow-up angiogram demonstrating durable complete aneurysm occlusion

**Figure 5:**
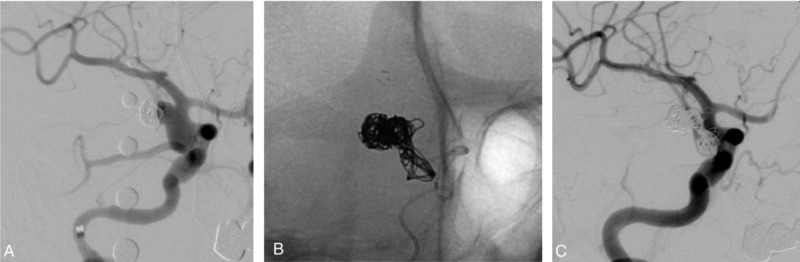
Female patient in her 70s treated with stent-assisted coil embolization for aneurysm recurrence after the previous coiling. (A) Angiogram demonstrating a residual bilobed wide-necked PComm aneurysm. (B) Stent-assisted embolization was performed by detaching the coils through the struts of the stent. (C) Post-procedure angiogram demonstrating dense coil packing and complete aneurysm occlusion

The intracranial stent facilitates the aneurysm thrombosis through several mechanisms. It provides protection for the parent vessel. It also allows increased packing density with low risk of coiling herniation, and it disrupts the aneurysm inflow (Figures 4-5).

Satisfactory occlusion of a complex aneurysm is challenging and there is an incomplete understanding of the treated aneurysms’ behavior over time. Initial occlusion in wide-necked aneurysms has been reported to range from 19 to 98% with an overall rate of 71% of complete occlusion at the latest imaging follow-up [15,21-24]. For statistical purposes we evaluated the probability of occlusion over time with an initial probability of complete occlusion estimated by the Kaplan-Meier analysis of 31.7% progressing to 88.5% at 48-month follow-up, which is similar to estimates performed by Santillan et al. in 79 wide-necked aneurysms treated with the Neuroform stent though their analysis included both ruptured and unruptured aneurysms [25]. They found an initial cumulative percentage of complete aneurysm occlusion of 42.2%, which progressed to 82.6% at 48 months and 96.5% at 84 months. Thus, our series is in accordance with the reported occlusion frequency and contributes to confirming the trend of progressive aneurysm occlusion over time. 

Due to the metal surface of stents, there is a high risk of thrombogenicity and dual-antiplatelet therapy plays an important role in preventing thromboembolic events. It should be given prior to the procedure and maintained for 3 to 6 months. The risk of latent bleeding should be considered when choosing SAC. Studies comparing SAC versus coiling alone or versus balloon-assisted coiling have demonstrated similar periprocedural complications, with lower rates of recurrence in the SAC group [25-28]. In the subset of wide-necked aneurysms, there is still controversy over long-term complications such as in-stent stenosis and recanalization when using SAC. In this regard, recent systematic reviews have reported a rate of in-stent stenosis ranging from 1.2 to 5.3% and recanalization rates ranging from 10 to 13.9% [4,16]. Our findings showed an overall rate of in-stent stenosis of 2.9%. Only one patient with severe in-stent stenosis (>50%) developed a transient ischemic attack and required balloon angioplasty. In similar studies, Fiorella et al. [29] reported a rate of in-stent stenosis of 5.6%, whereas Geyik et al. [20] recorded a rate as low as 0.8%. In contrast, Geyik et al. reported a recanalization rate of 10% and a retreatment rate of 7%, while Maldonado et al. [15] reported higher recanalization rate at latest follow-up of 17.5%. In comparison with the aforementioned studies, we found a recanalization rate of 6.6%. Two out of 5 aneurysms with recanalization retreated because they had evolved to Raymond-Roy class 3, while we decided to observe the aneurysms in class 2. In our study, the overall rate of aneurysms that required retreatment was 7.8% over the 5-year period. Based on these findings, we consider SAC a safe technique to treat unruptured wide-necked aneurysms with a low rate of long-term complications. 

Study limitations and generalizability

This study has some limitations. The design was observational with retrospectively collected data. In addition, results reflect a single-operator and single-center experience and results may not be generalizable to other centers. Regarding the secondary endpoints, in the time-to-event analysis, we assumed the aneurysm remained occluded if there was no further follow-up. However, further studies are required to elucidate the risk factors associated with the failure of occlusion. Stent-stenosis, recanalization, and retreatment were analyzed based on patients with at least one imaging follow-up. Additionally, SAC techniques and a variety of stents may contribute to the results but associations were not analyzed. Of note, 31 aneurysms have been previously reported [30].

## Conclusions

Stent-assisted coiling is a safe technique to treat unruptured wide-necked aneurysms in the endovascular armamentarium with a low rate of complications, stent-stenosis, and recanalization. Despite new technologies such as flow diversion or intrasaccular flow diverters, the SAC is still a safe alternative with favorable results at high-volume centers.

## References

[1] Guglielmi G, Vinuela F, Dion J, Duckwiler G (1991). Electrothrombosis of saccular aneurysms via endovascular approach. Part 2: preliminary clinical experience. J Neurosurg.

[2] Akpek S, Arat A, Morsi H, Klucznick RP, Strother CM, Mawad ME (2005). Self-expandable stent-assisted coiling of wide-necked intracranial aneurysms: a single-center experience. AJNR Am J Neuroradiol.

[3] Biondi A, Janardhan V, Katz JM, Salvaggio K, Riina HA, Gobin YP (2007). Neuroform stent-assisted coil embolization of wide-neck intracranial aneurysms: strategies in stent deployment and midterm follow-up. Neurosurgery.

[4] McLaughlin N, McArthur DL, Martin NA (2013). Use of stent-assisted coil embolization for the treatment of wide-necked aneurysms: A systematic review. Surg Neurol Int.

[5] Jabbour P, Koebbe C, Veznedaroglu E, Benitez RP, Rosenwasser R (2004). Stent-assisted coil placement for unruptured cerebral aneurysms. Neurosurg Focus.

[6] Roy D, Milot G, Raymond J (2001). Endovascular treatment of unruptured aneurysms. Stroke.

[7] Hanel RA, Taussky P, Dixon T (2014). Safety and efficacy of ticagrelor for neuroendovascular procedures. A single center initial experience. J Neurointerv Surg.

[8] Nordeen JD, Patel AV, Darracott RM (2013). Clopidogrel resistance by P2Y12 platelet function testing in patients undergoing neuroendovascular procedures: incidence of ischemic and hemorrhagic complications. J Vasc Interv Neurol.

[9] Mericle RA, Lanzino G, Wakhloo AK, Guterman LR, Hopkins LN (1998). Stenting and secondary coiling of intracranial internal carotid artery aneurysm: technical case report. Neurosurgery.

[10] Higashida RT, Smith W, Gress D, Urwin R, Dowd CF, Balousek PA, Halbach VV (1997). Intravascular stent and endovascular coil placement for a ruptured fusiform aneurysm of the basilar artery. Case report and review of the literature. J Neurosurg.

[11] Fiorella D, Albuquerque FC, Han P, McDougall CG (2004). Preliminary experience using the Neuroform stent for the treatment of cerebral aneurysms. Neurosurgery.

[12] Howington JU, Hanel RA, Harrigan MR, Levy EI, Guterman LR, Hopkins LN (2004). The Neuroform stent, the first microcatheter-delivered stent for use in the intracranial circulation. Neurosurgery.

[13] Henkes H, Bose A, Felber S, Miloslavski E, Berg-Dammer E, Kuhne D (2002). Endovascular coil occlusion of intracranial aneurysms assisted by a novel self-expandable nitinol microstent (neuroform). Interv Neuroradiol.

[14] Nishido H, Piotin M, Bartolini B, Pistocchi S, Redjem H, Blanc R (2014). Analysis of complications and recurrences of aneurysm coiling with special emphasis on the stent-assisted technique. AJNR A J Neuroradiol.

[15] Maldonado IL, Machi P, Costalat V, Mura T, Bonafe A (2011). Neuroform stent-assisted coiling of unruptured intracranial aneurysms: short- and midterm results from a single-center experience with 68 patients. AJNR Am J Neuroradiol.

[16] King B, Vaziri S, Singla A, Fargen KM, Mocco J (2015). Clinical and angiographic outcomes after stent-assisted coiling of cerebral aneurysms with Enterprise and Neuroform stents: a comparative analysis of the literature. J Neurointerv Surg.

[17] Kim SR, Vora N, Jovin TG (2008). Anatomic results and complications of stent-assisted coil embolization of intracranial aneurysms. Interv Neuroradiol.

[18] Gao X, Liang G, Li Y, Wu Z (2010). Neuroform stent-assisted coiling of large and giant intracranial aneurysms: angiographic and clinical outcomes in 71 consecutive patients. Neurol India.

[19] Kis B, Weber W, Berlit P, Kuhne D (2006). Elective treatment of saccular and broad-necked intracranial aneurysms using a closed-cell nitinol stent (Leo). Neurosurgery.

[20] Geyik S, Yavuz K, Yurttutan N, Saatci I, Cekirge HS (2013). Stent-assisted coiling in endovascular treatment of 500 consecutive cerebral aneurysms with long-term follow-up. AJNR Am J Neuroradiol.

[21] Sani S, Jobe KW, Lopes DK (2005). Treatment of wide-necked cerebral aneurysms with the Neuroform2 Treo stent. A prospective 6-month study. Neurosurg Focus.

[22] Weber W, Bendszus M, Kis B, Boulanger T, Solymosi L, Kuhne D (2007). A new self-expanding nitinol stent (Enterprise) for the treatment of wide-necked intracranial aneurysms: initial clinical and angiographic results in 31 aneurysms. Neuroradiology.

[23] Katsaridis V, Papagiannaki C, Violaris C (2006). Embolization of acutely ruptured and unruptured wide-necked cerebral aneurysms using the neuroform2 stent without pretreatment with antiplatelets: a single center experience. AJNR Am J Neuroradiol.

[24] Wajnberg E, de Souza JM, Marchiori E, Gasparetto EL (2009). Single-center experience with the Neuroform stent for endovascular treatment of wide-necked intracranial aneurysms. Surg Neurol.

[25] Santillan A, Greenberg E, Patsalides A, Salvaggio K, Riina HA, Gobin YP (2012). Long-term clinical and angiographic results of Neuroform stent-assisted coil embolization in wide-necked intracranial aneurysms. Neurosurgery.

[26] Hong Y, Wang YJ, Deng Z, Wu Q, Zhang JM (2014). Stent-assisted coiling versus coiling in treatment of intracranial aneurysm: a systematic review and meta-analysis. PLoS One.

[27] Yang H, Sun Y, Jiang Y, Lv X, Zhao Y, Li Y, Liu A (2015). Comparison of stent-assisted coiling vs coiling alone in 563 intracranial aneurysms: safety and efficacy at a high-volume center. Neurosurgery.

[28] Wang F, Chen X, Wang Y, Bai P, Wang HZ, Sun T, Yu HL (2016). Stent-assisted coiling and balloon-assisted coiling in the management of intracranial aneurysms: a systematic review and meta-analysis. J Neurol Sci.

[29] Fiorella D, Albuquerque FC, Woo H, Rasmussen PA, Masaryk TJ, McDougall CG (2010). Neuroform stent assisted aneurysm treatment: evolving treatment strategies, complications and results of long term follow-up. J Neurointerv Surg.

[30] Brasiliense LB, Yoon JW, Orina JN, Miller DA, Tawk RG, Hanel RA (2016). A reappraisal of anterior communicating artery aneurysms: a case for stent-assisted embolization. Neurosurgery.

